# Convergence Along the Visual Hierarchy Is Altered in Posterior Cortical Atrophy

**DOI:** 10.1167/iovs.61.11.8

**Published:** 2020-09-08

**Authors:** Pieter B. de Best, Ruth Abulafia, Ayelet McKyton, Netta Levin

**Affiliations:** 1 fMRI Unit, Neurology Department, Hadassah-Hebrew University Medical Center, Jerusalem, Israel

**Keywords:** complex visual problems, simultanagnosia, foveal crowding, fMRI, connective field, posterior cortical atrophy

## Abstract

**Purpose:**

Posterior cortical atrophy (PCA) is a rare neurodegenerative syndrome manifesting with visuospatial processing impairment. We recently suggested that abnormal population receptive field properties are associated with the symptoms of PCA patients. Specifically, simultanagnosia, the inability to perceive multiple items simultaneously, can be explained by smaller peripheral population receptive fields, and foveal crowding, in which nearby distractors interfere with object perception, may result from larger foveal population receptive fields. These effects occurred predominantly in V1, even though atrophy mainly involves high-order areas. In this study, we used connective field modeling to better understand these inter-area interactions.

**Methods:**

We used functional magnetic resonance imaging to scan six PCA patients and eight controls while they viewed drifting bar stimuli. Resting-state data were also collected. Connective field modeling was applied for both conditions: once when the source was V1 and the targets were extrastriate areas and once for the opposite direction. The difference between the two was defined as convergence magnitude.

**Results:**

With stimulus, the convergence magnitude of the controls increased along the visual pathway, suggesting that spatial integration from V1 becomes larger up the visual hierarchy. No such slope was found in the PCA patients. The difference between the groups originated mainly from the dorsal pathway. Without stimulus, the convergence magnitude was negative, slightly more so for the PCA patients, with no slope, suggesting constant divergence along the visual hierarchy.

**Conclusions:**

Atrophy in one part of the visual system can affect other areas within the network through complex intervisual area interactions, resulting in modulation of population receptive field properties and an ensemble of visuocognitive function impairments.

Posterior cortical atrophy (PCA) is a rare neurodegenerative syndrome involving high-order visual cortices. This relatively confined atrophy (at least in the early stages) results in impaired visuospatial processes despite normal visual acuity (agnosias). Often occurring visuospatial impairments include simultanagnosia and foveal crowding.^1^^,^^2^ Simultanagnosia, the impaired simultaneous perception of multiple items, is thought to result from spatially restricted vision.^3^ Paradoxically, foveal crowding, in which nearby distractors interfere with object perception, is thought to result from spatially extended processing.^4^

Novel neurocomputational modeling techniques provide insight into the neural correlates of these paradoxical symptoms. The population receptive field technique models the part of the visual field to which the neural populations in a brain volume unit (voxel) respond.^5^^–^^7^ Another approach is the connective field technique, which assesses how voxels in a target area (e.g., V2) are explained by a circular Gaussian that is folded to follow the cortical surface.^8^^–^^10^ These modeling approaches are usually based on functional magnetic resonance imaging (fMRI) data that have been acquired under visual stimuli conditions. Another way to study connectivity is by examining spontaneous neural activity, during resting state conditions (RS-fMRI). In this approach, as well, connective field modeling predicts the activity of voxels in one visual area as a function of the aggregate activity in voxels in another visual area, but in the absence of visual input. Connective field size estimates obtained for RS-fMRI are generally smaller than those obtained under visual stimuli conditions and tend not to increase throughout the visual hierarchy as stimuli-based connective fields do.^10^

An important aspect of connective field modeling is that it provides information about the direction of information flow in terms of convergent versus divergent connections. If connective field sizes in the upstream flow of information are larger than those in the downstream direction, this indicates that visual information converges up the visual processing cortical hierarchy.^9^

We recently applied the population receptive field model to find indications that simultanagnosia can be explained by smaller population receptive fields at high eccentricity.^11^ Additionally, we found that foveal crowding may result from larger foveal population receptive fields. These effects occurred predominantly in V1, even though cortical atrophy mainly affects extrastriate visual areas.

Based on these results, we suggested that altered V1 population receptive field sizes are attributed to a combined mechanism by which atrophy of high-order association cortices simultaneously causes impairment of attention processes and a disruption of basic visual processes via altered feedback. This feedback connection interference (due to atrophy of higher visual regions) could result in modulation of the population receptive field properties in V1. Yet, these changes in basic cortical characteristics are clinically manifested as a grouping of high-order visuocognitive functions.^11^ Herein, we have used state-of-the-art connective field modeling protocols to investigate how high-order visual impairment may result from altered intervisual area interactions.

## Methods

### Subjects

Six PCA patients (mean age ± SD, 64.6 ± 8.2 years; one female) were studied. PCA was diagnosed based on the criteria described in the meta-analysis of Alves et al.^12^ and the first two levels of the consensus criteria described by Crutch et al.^13^ Average time from initial symptoms was 2.7 ± 0.8 years. Eight age-matched healthy volunteers (age, 63.7 ± 10.5 years; seven females) were enrolled as a control group. All subjects had normal or corrected-to-normal visual acuity, which was tested using a computerized Snellen chart. The acuity threshold was set at 6/9 for each eye. Letters were presented in single-line format. When crowding was disruptive, testing was done one letter at a time. The Hadassah-Hebrew University Medical Center Ethics Committee approved the experimental procedure, and the study was conducted in accordance with the tenets of the Declaration of Helsinki. Written informed consent was obtained from all subjects.

### Data Acquisition

fMRI data were acquired using a Siemens 3T MAGNETOM Skyra scanner (Siemens Medical Solutions, Malvern, PA, USA). To acquire 20 coronal functional slices covering the visual areas, the posterior part of the 32-channel receiver coil was used to perform two-dimensional echo-planar imaging sequences, with repetition time (TR)/echo time (TE) = 1500/27 ms, flip angle = 55°, isotropic voxel size = 2.5 mm, and field of view = 180 × 180 mm. In-plane anatomical scans (TR/TE = 300/3.78 ms, flip angle = 60°, voxel size = 0.8 × 0.8 × 2.5 mm; 20 coronal slices, 200 × 200 mm) were performed after each viewing condition. The full 32-channel coil was used to perform a high-quality magnetization-prepared rapid acquisition with gradient echo (MPRAGE) anatomy scans, where TR/TE = 2300/2.98 ms, flip angle = 9°, isotropic voxel size = 1 mm, and 160 axial slices (256 × 256 mm) covered the whole brain. Resting-state fMRI measurements (8-minutes scans) were obtained with an echo-planar imaging sequence, where TR/TE  =  2000/30 ms, flip angle = 90°, and isotropic voxel size = 3 mm, with 32 slices (192 × 192 mm).

For the stimulation connective field experiment, we used 2° wide bar apertures that revealed a moving checkerboard pattern in 16 steps of 1° and 1.5 seconds (high-contrast, 0.5 cycle per degree spatial frequency). The pattern moved parallel to the bar orientation. The width of the bar subtended one-fourth of the stimulus radius (8°). Four bar orientations (0°, 45°, 90°, and 135°) and two different motion directions for each bar orientation were used, resulting in eight different bar directions within a given scan. Participants reported color changes of a fixation dot.^6^

The VISTADISP toolbox (GitHub, https://github.com/vistalab/vistadisp) and Psychtoolbox^14^ were used for stimulus creation. Stimuli were transferred through a mirror converter box to a 32-inch MR-compatible liquid-crystal display monitor (NordicNeuroLab, Bergen, Norway), which was placed at a 140-cm viewing distance. Stimuli were projected onto a mirror placed above the subject's head.

### Data Preprocessing

Functional data preprocessing included removal of eight functional pre-scan volumes, slice timing correction, and within- and between-scan motion compensation.^15^ The MPRAGE scans were realigned in the anterior commissure (AC)–posterior commissure (PC) space through AC and PC identification. An automatic gray–white matter segmentation was performed with Freesurfer^16^ and manually adjusted. The in-plane scans were aligned with the MPRAGE scan using Nestares alignment^15^ and with Statistical Parametric Mapping (SPM) mutual information (http://www.fil.ion.ucl.ac.uk/spm/software/spm8/).

### Data Analysis

Population receptive field and connective field models were generated using VISTASOFT (GitHub, https://github.com/vistalab/vistasoft), according to Haak et al.^9^ and Gravel et al.^10^ After the population receptive fields were modeled, eccentricity and polar angle maps were derived from them and used to delineate the primary visual cortex (V1), V2d, V2v, V3d, V3v, human V4 (hV4), and object-associated (lateral–occipital, LO) and motion-associated (temporal–occipital, TO) areas, as described by de Best et al.^11^ The number of voxels representing the cortical surface area was compared between groups, with no significant differences being found between cohorts. For connective field modeling, the activity of a voxel in the target area (e.g., V2) was predicted by blood oxygen-level dependent (BOLD) activity in the cortical surface of the source area (e.g., V1), termed V1 to V2 connective fields, as described in detail in Haak et al.^9^

Briefly, the preprocessed BOLD responses were first high-pass filtered by removing the linear trend from the data. The reason for choosing only high-pass filtering for this dataset was to avoid filtering out the stimulus-associated signal along with the noise. Specifically, filtering from 0.01 to 0.1 Hz would mean that the parts of the signal that happen outside this time range would be filtered out. The target area BOLD response was then predicted by folding a circular Gaussian connective field model, defined by position (*v*_0_) and size (σ), over the cortical surface of the source region of interest and calculating the weighted sum of voxel responses. For each voxel, these parameters were adjusted until the error between the model-predicted and the observed fMRI time-series was minimized. The connective field size was corrected for ipsilateral visual representations and was termed sampling extent, as in Haak et al.^9^

Similarly, these connective field models were also applied to resting-state fMRI scans; however, these scans were bandpass filtered to include frequencies between 0.01 and 0.1 Hz, rather than just high-pass filtered, in order to clean out both baseline drift and high-frequency physiological noise, as described by Gravel et al.^10^ Classical resting-state data cleaning procedures usually involve more steps, such as independent component analysis-based denoising of the white matter and cerebrospinal fluid in order to reveal small functional differences among areas that could be otherwise hidden. In connective field modeling, however, frequencies that are shared among white matter, cerebrospinal fluid, and cortex might contribute to the connective field measurements; therefore, we did not discard them.

For both stimulation and resting state-based connective fields, we assessed V1 to V2/V2v/V2d/V3/V3v/V3d/hV4/LO/TO (i.e., V1 to region *x*) and V2/V2v/V2d/V3/V3v/V3d/hV4/LO/TO to V1 connective fields (i.e., region *x* to V1). In these models, voxels with ≥20% variance explained were further analyzed. Connective field sizes were searched by the model in the following descriptive steps: 0.0001 mm, 0.2 mm, 0.4 mm, 0.6 mm, and so on until 10 mm. When 0.0001 mm is chosen (fulfills the role of 0), that means that all other higher values explained less variance and neighboring voxels did not contribute to the model; that is, neighboring voxels did not have any shared response that could be detected (combining them into a surface). This indicates that there was no “connective field area,” only a one-on-one connection; thus, connective fields with sizes smaller than or equal to 0.0001 mm were excluded. Median sampling extents, weighted by variance explained, were calculated over eccentricities 0.5° to 7.5°. The median connective field size was calculated as such because connective field size was reported to be constant across eccentricities within an area.^9^

The differences between the sampling extents of V1 to region *x* and region *x* to V1 were calculated to indicate the convergence magnitude. Here, higher values indicate a larger spatial extent, whereas positive and negative values, respectively, indicate convergence and divergence from V1 to region *x*. The two-sided Wilcoxon rank-sum test was applied to convergence magnitudes for comparisons between PCA patients and controls regarding the connections between V1 and the nine extrastriate areas. Bonferroni multiple-comparisons correction was applied for these nine comparisons.

We also assessed convergence magnitude along the visual hierarchy. In order to do so, we derived hierarchical levels from Haak et al.^17^^,^^18^ in order to give some ordinal value to each visual area. In these studies, V1, V2, V3, hV4, LO1, LO2, TO1, and TO2 were, respectively, at hierarchical levels 0, 1, 2, 3, 3, 4, 5 and 6. Because we assessed LO1/LO2 and TO1/TO2 together (referred to as LO and TO in the rest of the text), we assigned them the average values of, respectively, 3.5 and 5.5. We assessed these levels in both streams together (i.e., V1 ↔ V2, V3, hV4, LO, and TO), in the dorsal stream (i.e., V1 ↔ V2d, V3d, and TO), and in the ventral stream (i.e., V1 ↔ V2v, V3v, hV4, and LO). For each participant, convergence magnitude was linearly regressed using these levels to assess the slope of convergence magnitude along the visual hierarchy. We assessed whether there was a slope in each group (using a single-sample signed-rank test to compare it to 0) and whether it differed between patient and control groups (using the Wilcoxon rank-sum test). *P* values were Bonferroni corrected for the three comparisons (both streams, dorsal stream, and ventral stream).

We compared differences between V1 to region *x* and region *x* to V1 in goodness of fit, number of voxels that were included in the assessment (after excluding connective field sizes of 0.0001 or smaller; referred to as number of voxels in the results), and target area eccentricity between PCA patients and controls, as changes in these variables could potentially bias the results. If a difference was found, we assessed the Spearman correlation coefficient between the biasing variable and the convergence magnitude. Here, too, Bonferroni correction was used.

## Results

**Figure 1.**
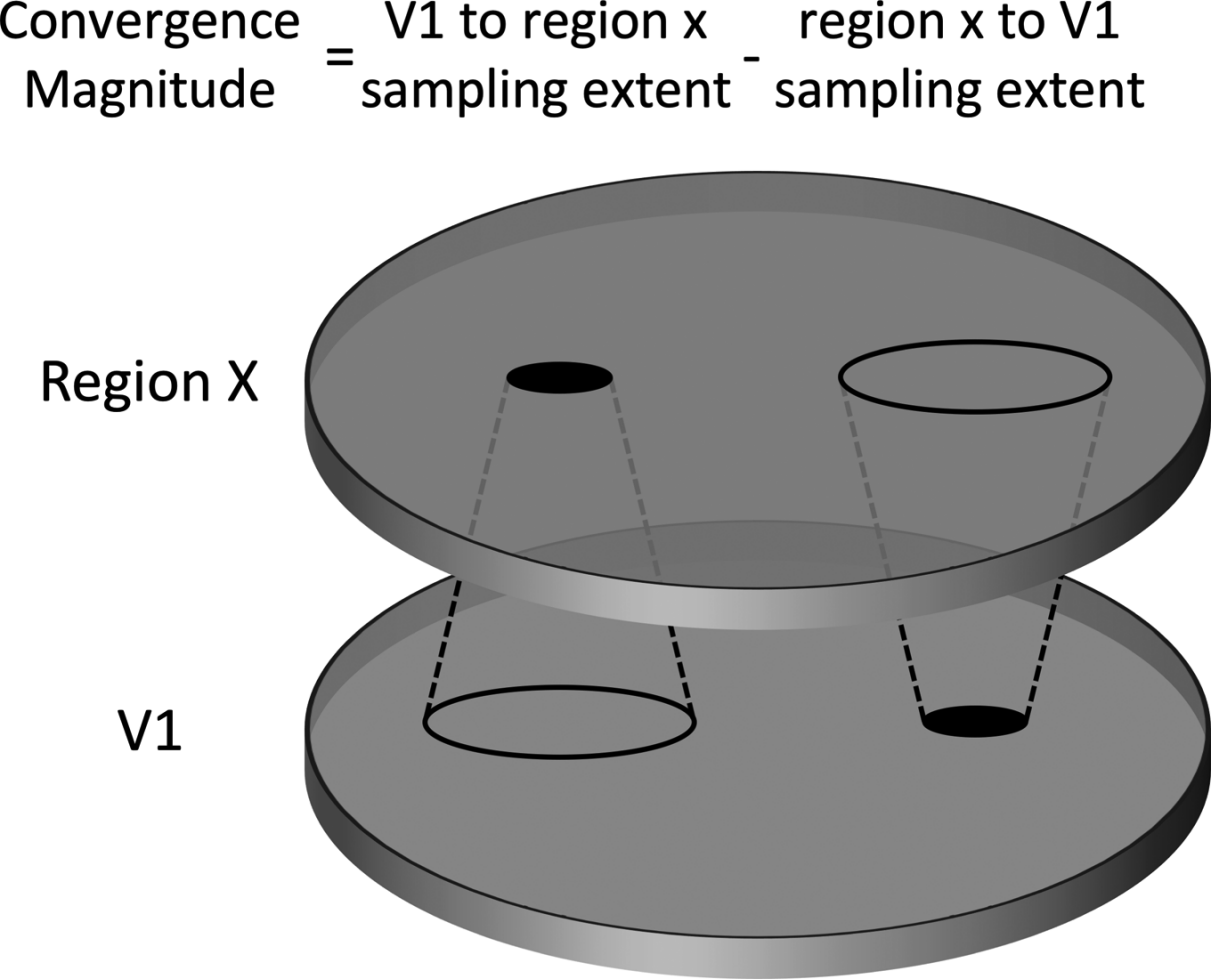
Convergence magnitude. V1 to region *x* sampling extent is the circular area in V1 that was sampled by a single voxel in (extrastriate visual) region *x*, and vice versa for region *x* to V1 sampling extent. Convergence magnitude is defined as the difference between the two.

Motion parameters were compared between groups for both the resting state and stimulus conditions, and no significant differences were found in either of them. The data extracted were used to calculate sampling extents in which the source area was V1 and the target areas were extrastriate areas (Fig. 1). The opposite direction (extrastriate areas as source and V1 as target) was also calculated (Fig. 1). The difference between the two, which indicates convergence,^9^ was used for analysis and was termed convergence magnitude.

### Stimulus-Based Sampling

**Figure 2.**
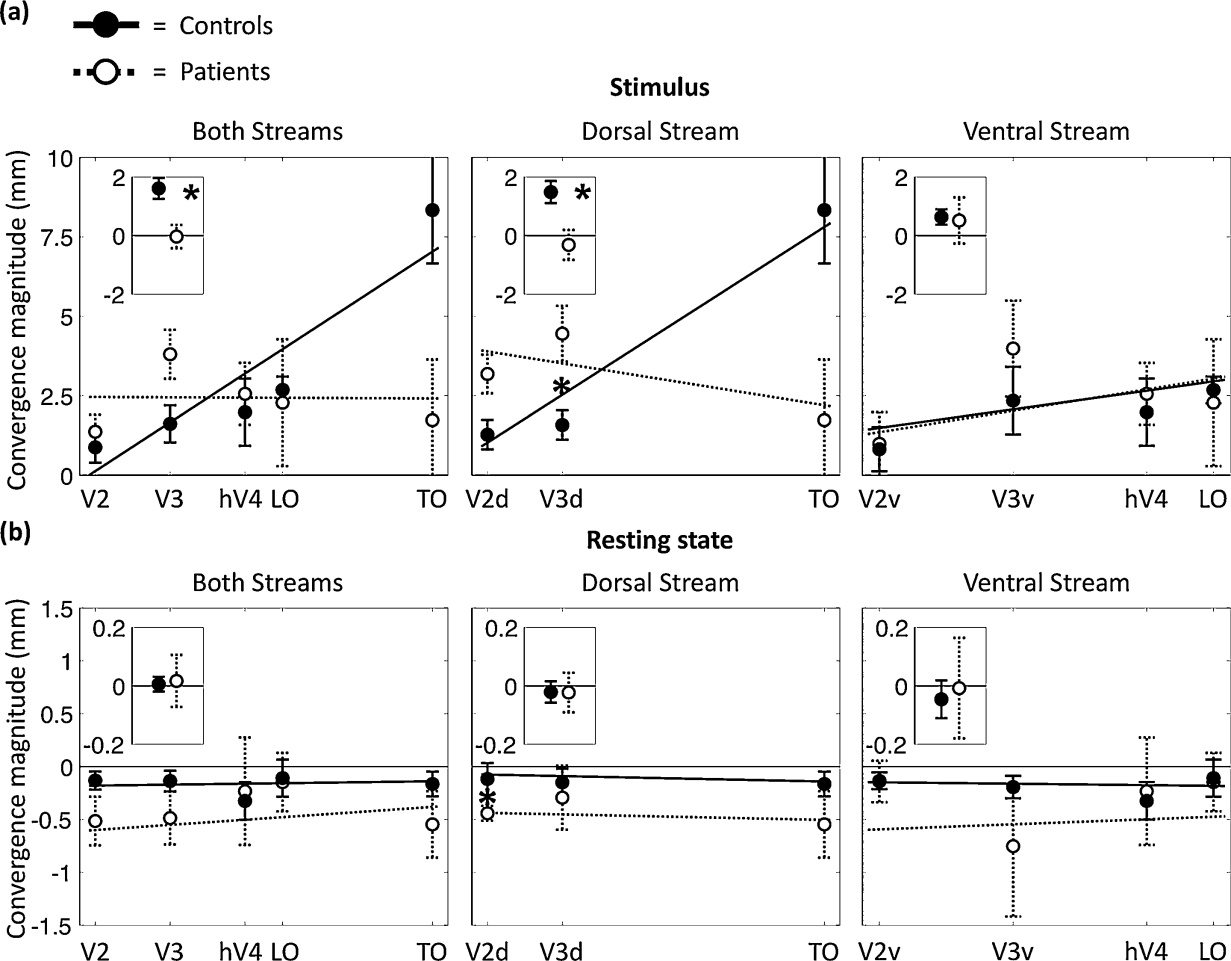
Convergence magnitude in the visual system. Stimulus-based and resting-state-based convergence magnitudes are shown for PCA patients (empty circles) and controls (filled). The different visual areas were positioned along the *x*-axis using their hierarchical levels.^17^^,^^18^ In the main graphs, a significant difference between groups in a specific region is denoted by an asterisk (*P* < 0.05, corrected for multiple comparisons). Linear trend lines were fitted to the data for each individual and were used for statistical comparisons between groups (inlay graphs). Asterisks inside the inlay graphs denote significant differences in slopes between groups (*P* < 0.05, corrected for multiple comparisons). For visualization purposes, linear trend lines were also calculated using the individual values from each group and are presented in the main graphs. Error bars represent standard error of the median.

When a stimulus was used, convergence magnitude was positive for both PCA patients and controls, suggesting convergence from V1 to extrastriate areas (Fig. 2a). As expected, convergence magnitude in the controls followed a positive slope along the visual pathway (median ± standard error of the median, 1.61 ± 0.36; W = 36; *P_c_* = 0.024, corrected for multiple comparisons), meaning that spatial integration from V1 became larger going up in the hierarchy. To verify that this result was not driven by visual area TO alone, we repeated the analysis without TO and revealed a similar effect (0.92 ± 0.22; W = 35; *P_c_* = 0.047). This does not appear to have been the case among the PCA patients (–0.035 ± 0.40; W = 10; *P_c_* = 1), who differed significantly from controls (W = 80; *P_c_* = 0.024). When dividing the visual pathway into ventral and dorsal streams, it seems that the difference between the groups originated from differences along the dorsal pathway (dorsal stream: controls, 1.49 ± 0.38, PCA, –0.32 ± 0.51, W = 82, *P_c_* = 0.009; ventral stream: controls, 0.63 ± 0.26, PCA, 0.51 ± 0.79, W = 60, *P_c_* = 1).

Specifically, the convergence magnitude between V1 and V3d was larger in the PCA patients as compared to controls (4.45 ± 0.88 and 1.58 ± 0.46 mm in PCA and controls, respectively; W = 39; *P_c_* = 0.045). The convergence magnitude between V1 and visual area TO was also larger in PCA patients as compared to controls; however, this difference did not reach significance after correcting for multiple comparisons (1.73 ± 1.91 and 8.34 ± 1.68 mm in PCA and controls, respectively; W = 79; *P_c_* = 0.11).

To evaluate potential biasing variables, goodness of fit, number of voxels, proportion of excluded voxels (because connective field [CF] = 0.0001 mm), and eccentricity were compared between PCA patients and controls. No differences in these variables were found to underlie the difference found in convergence magnitude between V1 and V3d (four variables). Our evaluation of the goodness of fit for all areas showed that, as in the controls, the PCA patients had reasonably robust stimulus-driven activity in the areas tested (Table). Additionally, we calculated the difference in number of voxels between V1 to region *x* and region *x* to V1. PCA patients and controls did not differ in the slope of this value along the hierarchy.

**Table.** Goodness of Fit^*^

	Stimulus, Variance Explained (Median ± Standard Error of the Median)	
Connective Field	PCA Patients	Controls
V1 to V2	0.84 ± 0.05	0.84 ± 0.02
V2 to V1	0.83 ± 0.06	0.85 ± 0.02
V1 to V2d	0.84 ± 0.04	0.84 ± 0.02
V2d to V1	0.81 ± 0.05	0.82 ± 0.03
V1 to V2v	0.80 ± 0.06	0.82 ± 0.02
V2v to V1	0.74 ± 0.07	0.78 ± 0.03
V1 to V3	0.83 ± 0.07	0.82 ± 0.02
V3 to V1	0.79 ± 0.08	0.82 ± 0.03
V1 to V3d	0.85 ± 0.07	0.81 ± 0.03
V3d to V1	0.78 ± 0.08	0.80 ± 0.03
V1 to V3v	0.78 ± 0.06	0.80 ± 0.02
V3v to V1	0.72 ± 0.08	0.73 ± 0.03
V1 to hV4	0.63 ± 0.06	0.67 ± 0.03
hV4 to V1	0.61 ± 0.07	0.65 ± 0.02
V1 to LO	0.63 ± 0.05	0.70 ± 0.03
LO to V1	0.65 ± 0.06	0.66 ± 0.03
V1 to TO	0.44 ± 0.07	0.52 ± 0.04
TO to V1	0.46 ± 0.07	0.49 ± 0.04

* Goodness of fit is measured through variance explained in proportion (i.e., values from 0 to 1 that would represent 0% to 100%) in the stimulus conditions.

### Resting State-Based Sampling

Without stimulus, median convergence magnitudes appeared to be negative for both PCA patients and controls, without any slope between areas, suggesting similar divergence from V1 to the different extrastriate areas. This divergence seemed stronger for the PCA patients than controls, with a significant difference in V2d (PCA, –0.44 ± 0.07; control, –0.12 ± 0.15 mm; W = 81; *P_c_* = 0.045). No significant differences in the intercepts of the regression lines were found between groups: for both streams, W = 69 and *P_c_* = 0.84; for the dorsal stream, W = 76 and *P_c_* = 0.13; and for the ventral stream, W = 73 and *P_c_* = 0.33.

Here, too, we evaluated the potential biasing variables (goodness of fit, number of voxels, proportion of CF = 0.0001 or eccentricity biases) between PCA patients and controls. In assessing the difference in number of voxels between V1 to V2d and V2d to V1, we found that this difference was larger in PCA patients than in controls (respectively, –609 ± 63 and –239 ± 60 voxels; W = 82; *P_c_* = 0.027). We also found a difference in the proportion of CF = 0.0001 mm (for PCA patients and controls, respectively: 0.16 ± 0.04 and –0.11 ± 0.04; W = 38; *P_c_* = 0.027). However, no correlation was found between these differences and the convergence magnitude (respectively, Spearman's ρ = 0.50, *P_c_* = 0.07; Spearman's ρ = –0.47, *P_c_* = 0.09).

## Discussion

We found evidence, using PCA as a model, that atrophy in one part of the visual system can affect other areas within the network through complex intervisual area interactions. Using stimulus-based connective field models, convergence from V1 to extrastriate areas was evident in both controls and PCA patients, but the spatial extent of integration did not increase along the hierarchy in the PCA patients as in controls. This indicates that high-order dorsal stream areas may receive information from a more spatially restricted area in PCA patients, which could explain simultanagnosia (where multiple objects do not fit in the spatially restricted field of information visual area TO receives). On the other hand, V3d, which appears to integrate spatial information from a larger area in V1, may do so in order to compensate for undersampling of V1 by the visual area TO.

Using resting state-based connective field models, a diverging pattern, similar among visual areas along the hierarchy, was evident in both controls and PCA patients. The spatial extent of this diverging pattern seemed larger in PCA patients, reaching significance in area V2d. We suggest that these altered interactions may weaken V1 spatial resolution, resulting in the atypical phenomenon of foveal crowding.

Connective field modeling was first developed in 2013 by Haak et al.^9^ to describe the interaction between source and target areas within the visual network and to infer from these interactions the flow direction in terms of convergent or divergent connectivity. For example, if V1 to V2 connective fields are larger than those of V2 to V1, convergence from V1 to V2 exists (which is presumably usually the case upstream in the visual system). Indeed, in our normal-sighted control cohort, under visual stimulus conditions visual information converged from V1 to high-order areas. Furthermore, the spatial magnitude of this convergence exhibited a positive slope, reflecting increasing spatial integration along the hierarchy. Although visual cortical processing is known to involve feedforward, horizontal, and feedback connections, it has been suggested that in the presence of visual stimulation feedforward brain activity dominates.^19^ Thus, connective fields that were modeled with visual stimulation were considered to be less sensitive to feedback influences.^8^

As opposed to visual stimulus-based cortical mapping, the control subjects’ connective field modeling in resting-state conditions indicated diverging patterns, suggesting cumulative top-down effects in the absence of visual stimuli. This finding corresponds with what was suggested by Halbertsma et al.^8^ Unlike visual stimulation conditions, the spatial convergence magnitude of the resting-state connective field does not change along the hierarchy. This was already found by Gravel et al.,^10^ who assessed V1 to V3 resting-state connective fields, but in this study we expanded this notion to high-order visual areas, as well.

Connective field modeling was recently used to try to expand our insights into perceptual phenomena and neuro-ophthalmological disorders.^8^^,^^20^^–^^25^ Following our previous work, in which we used stimulus-referred population receptive field models in PCA patients,^11^ here we attempted to use this novel imaging method to untangle unanswered questions. Briefly, the main finding in our previous work was that the foveal population receptive fields of PCA patients were larger than those of controls, whereas the peripheral population receptive fields were smaller.^11^ The puzzling part was that these alterations were more pronounced in early visual areas, although pathology and cortical atrophy generally characterize extrastriate visual areas in PCA patients.^26^^–^^35^ Thus, we hypothesized that the observed population receptive field changes were a consequence of a disruption in the delicate balance between excitatory and inhibitory signals in V1 through impaired feedback influences.

The results of the current study suggest that simultanagnosia may be explained by the notion that V1 is being undersampled in high-order visual areas during stimulus presentation. By sampling a smaller area on the surface of V1, TO potentially receives inputs from a significantly smaller area, which could be in line with the suggested shrinkage of spatial attention^3^ and impaired parallel object processing^36^ as potential underlying factors of simultanagnosia. This hypothesis fits with evidence from lesion studies suggesting the dorsal stream as the dominant pathway for global processing. In this context, damage to one of the core regions along the dorsal stream, such as TO, may disrupt the processing of the coarse, low-spatial frequency information that is critical for generating a global picture, giving rise to facilitation of local processing via the ventral stream, that is characteristic of simultanagnosia.^37^ The relationship between TO dysfunction and various symptoms that are traditionally related to the ventral stream, such as reading, has previously been suggested. For example the role of the visual word form area in acquired dyslexia was suggested to be part of a more general deficit in simultaneous visual processing through its functional correlations with regions in the dorsal attention network (left and right anterior intraparietal sulcus, middle temporal complex, and frontal eye field regions).^38^ White matter connected to motion areas was also suggested as a possible causal factor for simultanagnosia. Localized white matter atrophy and impaired integrity in the vicinity of PCA patients’ motion-related areas were correlated to the slowing of visual processing speed and clinical symptoms of simultanagnosia.^39^

Foveal crowding, on the other hand, could be mediated by altered feedback connectivity. Crowding, which is a breakdown in the ability to identify cluttered objects that majorly constrains object recognition,^4^ has been suggested to reflect pooled target and distracting stimuli within the same receptive field.^40^ Thus, by receiving information from a larger area on the surface of upstream visual areas, V1 may receive information of both targets and distractors from visual space, resulting in foveal crowding. In line with our findings, He et al.^41^ recently reported that in normally sighted subjects larger V2 population receptive field sizes are predictive of the magnitude of visual orientation crowding. Additionally, two studies in primates demonstrated changes in V1 receptive field properties following inactivation of V2/V3 cortical areas,^42^^,^^43^ supporting the notion that feedback interactions could affect receptive field extents.

Finally, we found that V3d appears to sample from a larger area in V1, perhaps in order to compensate for the lack of spatial integration in high-order areas (being a mid-hierarchical region downstream of the affected areas). A recent resting-state functional connectivity study by Haak et al.^17^ reported a complex relationship between visual network plasticity and the hierarchical level visual region, such that plasticity decreases from V1 to V3 and then increases further along the hierarchy. It is suggested that this non-monotonic function is a reflection of a combination of decreasing transient adaptive changes combined with increasing long-term plasticity along the hierarchy. It is important to note that, while ventral areas seem to be defined by transient changes alone, dorsal areas appear to be defined by both transient and long-term plasticity.

The fact that the difference between PCA patients and controls mainly originated from the dorsal pathway is not surprising. First, from a neural degeneration point of view, a functional deficit in the higher dorsal stream processing areas was previously suggested to dominate PCA clinical presentation.^44^ Furthermore, localized gray matter atrophy, local involvement of white matter fibers in the proximity to motion-related cortical areas,^39^ and high densities of senile plaque and neurofibrillary tangles in the MT area that were previously reported in pathological studies^30^ signify the specific role of the dorsal stream in the disease.

Second, from a perceptual learning point of view, the possible compensation mechanism we found between V1 and V3d could be explained by the results of human perceptual learning studies that have provided evidence for long-term plasticity in adult V1 to V3^45^^–^^48^ and in visual area TO.^45^^,^^46^^,^^49^ In general, areas along the ventral occipital surface tend to exhibit less plasticity than areas on the dorsal occipital surfaces.^17^^,^^50^^,^^51^ A possible explanation lies in the fact that dorsal visual areas are more involved in interacting with the environment, whereas ventral areas implement more stable integration codes.

This study is limited by the small number of PCA patients who were included due to the rarity of the disease. Additionally, differences in the cohorts’ gender could potentially result in gender-associated differences in cortical volume. However, the median surface of all visual areas did not differ between groups and therefore cannot explain the reported results.

## Conclusions

We previously suggested that PCA could provide a human model of feedback connection interference due to atrophy of high-order visual regions.^11^ The resting state fMRI branch of the current study revealed a trend for oversampling from the surface of extrastriate areas by V1 voxels. Given that resting state fMRIs are less dominated by the feedforward connections that are normally initiated by PCA patients viewing stimuli, we suggest that this could mean that, in PCA, larger parts of the extrastriate cortical surfaces send information to V1 voxels. This would, in turn, potentially lead to oversampling of visual space, explaining foveal crowding. In the stimulus-based fMRI, the lack of increase in the convergence magnitude along the visual hierarchy could, in turn, reflect altered feedforward connections. Potentially, the atrophy in visual area TO affects its input layers most. Undersampling in visual area TO could lead to undersampling of visual space and explain simultanagnosia. Finally, oversampling from the V1 surface by V3d may reflect a compensation attempt for these changes in visual area TO. In conclusion, combined bottom-up stimulus-based and top-down resting-state-based connective fields appear to be useful models in explaining the complex visual processing problems in PCA, as well as potentially complex visual processing issues stemming from other high-order visual disorders.
